# Childhood Obesity: Review of a growing Problem

**DOI:** 10.5005/jp-journals-10005-1175

**Published:** 2012-12-05

**Authors:** Anubhav Shivpuri, Abhay Shivpuri, Sunil Sharma

**Affiliations:** Ex Senior Lecturer, Department of Oral and Maxillofacial Surgery Mahatma Gandhi Dental College and Hospital, Jaipur, Rajasthan India, e-mail: dranubhavshivpuriomfs@gmail.com; Postgraduate Student, Department of Pediatrics, Bangalore Baptist Hospital, Bengaluru, Karnataka, India; Professor and Head, Department of Oral and Maxillofacial Surgery Mahatma Gandhi Dental College and Hospital, Jaipur, Rajasthan, India

**Keywords:** Obesity, Overweight

## Abstract

The consequences of obesity in adulthood are well known. Obesity has a direct influence on mortality and acts as a risk factor for various diseases and health problems. It is associated with nonfatal but debilitating illnesses, such as respiratory difficulties, musculoskeletal disorders, skin problems and infertility. The association with fatal chronic diseases includes cardiovascular diseases, conditions related to insulin resistance and noninsulin-dependent diabetes. There has been a marked increase in the number of obese children coming for treatment to dentists, thus it is the moral responsibility of the dentists to educate both the patient and the parents of the problems of obesity and its control. A dentist may actually be the first person to inform the patient about this problem thus, a basic knowledge about it is important.

**How to cite this article:** Shivpuri A, Shivpuri A, Sharma S. Childhood Obesity: Review of a growing Problem. Int J Clin Pediatr Dent 2012;5(3):237-241.

## INTRODUCTION

In the Indian culture it is considered good, if the child is well fed and a bit on the heavier side.^1-7^ Sometimes this obsession takes a wrong turn and the child ends up obese. The parents may not even realize that their child is obese thus do not consult a doctor which may lead to major health concerns as he/she grows. A dentist may be the first doctor who can diagnose and educate them about this problem when they consult him regarding dental check-up. Thus, it is important that dentists should have basic knowledge about obesity and its management.

The most recent estimates from the World Health Organization and the International Obesity Task Force of the International Association for the Study of Obesity are that there are more than 155 million children and adolescents around the world who are overweight, with approximately 40 million who are clearly obese.

Over the past 30 years, the prevalence of obesity has nearly tripled for children 2 to 5 years of age and it has quadrupled for children 6 to 11 years old.

Data from National Health and Nutrition Examination Surveys (1976-1980 and 2003-2004) show that for children 2 to 5 years of age, the prevalence of overweight increased from 5 to 13.9%; for those 6 to 11 years of age, prevalence increased from 6.5 to 18.8%.

Overweight children and adolescents are at risk for significant health problems both during their youth and as adults. Overweight children are more likely than other children to have risk factors associated with cardiovascular disease (e.g. high blood pressure, high cholesterol and type 2 diabetes mellitus). Overweight children are also more likely to become obese as adults.

## CHILDREN AT RISK

Children who have obese parents, urban, economically underprivileged children, high calorie diet, children who are inactive and who overeat to cope with stress are generally more at risk.

## DEFINITION

Obesity can be defined as excess body fat in an individual. It is considered a chronic, noncommunicable disease.^8-10^ Such diseases are currently the principal causes of death in both developed and developing countries, thus making them one of the largest public health problems. Currently, ‘overweight' is defined by a body mass index (BMI)-for age of ≥85th percentile but <95th percentile in children and adolescents, and ‘obesity' is defined as a BMI-for age of ≥95th percentile.

## CAUSES

The cause of childhood obesity is certainly debated.^11,12^ Some researchers have pointed to socioeconomic factors, while others have accused mass media as the culprit for marketing junk food to children. Food makers have blamed physical inactivity and a lack of parent influence on diet. All these key factors have likely worked together to increase the prevalence of childhood overweight and obesity.

Abuse, anxiety, depression and family stress may be associated with obesity, potentially through the adrenal axis and/or stress-related eating patterns.

Limited physical activity during and after school hours contributes to childhood obesity.

Imbalance between energy intake and expenditure, genetic and environmental factors are equally important.

It has been estimated that 25 to 70% of the body weight variation can be attributed to genetics.

Although studies suggest that the genetic background must affect one or more component of energy balance, the mechanism by which genes may contribute to differences in body weight is less clear. Studies have reported that a low thermic effect of food, or an inability to oxidize fat with a high reliance on carbohydrate as a fuel may predispose to obesity.

In humans, autosomal recessive mutations in the genes for leptin, the leptin receptor, prohormone convertase 1 (PC1), and POMC, have been shown to lead to early onset obesity.

*Pathologic causes:* Hypothyroidism, Cushing's syndrome, leptin deficiency, etc.

*Drugs:* Few drug also lead to obesity, e.g. lithium, cortisone, valproate, mood stabilizers (elavil, xerostat), antipsychotics (chlorpromazine, ergenyl), migraine medicines (sandomigran), etc.

## PATHOPHYSIOLOGY OF OBESITY

The fundamental cause of obesity is a greater imbalance between energy intake and expenditure than is expected for normal growth and development.^6,13^ Usually, this occurs over a period of time and in the presence of a susceptible genetic background and environmental factors. Epigenetic factors, defined as the changes in gene function that do not relate to changes in DNA sequence, begin *in utero* also contribute. Infants of diabetic mothers and of mothers who smoke during pregnancy have increased risk of subsequent obesity. Infant feeding practices may also play a role, particularly a shortened period of breastfeeding. A reduced amount of sleep during infancy is another potential risk factor for obesity. Some medications have been clearly demonstrated to cause excess weight gain.

## CONSEQUENCES OF CHILDHOOD OBESITY

The increasing prevalence and severity of obesity in children and adolescents have resulted in a higher prevalence of comorbid conditions, including high blood pressure, early development of atherosclerosis, type 2 diabetes mellitus, nonalcoholic fatty liver disease, polycystic ovary disorder and disordered breathing during sleep.^6,14^ These compli- cations can occur both in the short-term and in the long- term. Some complications, such as type 2 diabetes mellitus, previously thought to only occur in adulthood have now been shown to occur in children and adolescents. The obesity epidemic might shorten the life span of the current generation of children.

## PRESENTATION

Gaining weight rapidly, awkward appearance, lethargic, breathless on exertion, snoring, small genital size or prominent breasts in boys comprises the typical appearance of an obese child.

Compared with children at a normal weight, overweight children are 70 to 80% more likely to be overweight in adulthood.

## CLINICAL EVALUATION

Evaluation of obesity begins with calculation of BMI, which has clinical validity because it correlates with adiposity, adult adiposity, cardiovascular risk factors and long-term mortality.^6,15-20^

Current definitions use the 85th percentile to define overweight and the 95th percentile to define obesity.

Details of diet and eating habits, activity patterns, duration of TV viewing, mental development, school performance, height, weight, abdominal/hip circumference, fat distribution are necessary for finding the root cause.

The increasing prevalence of obesity in children seems to be associated with an increased prevalence of obstructive sleep apnea syndrome (OSAS) in children. Possible pathophysiological mechanisms contributing to this association include the following: Adenotonsillar hypertrophy due to increased somatic growth, increased critical airway closing pressure, altered chest wall mechanics and abnormalities of ventilatory control.

## INVESTIGATIONS

### Guided by Clinical Presentation

A biochemical prole and a full blood count are useful as a baseline.^21^ Fasting plasma glucose and lipid prole should be done to exclude diabetes and dyslipidemia and serum- free thyroxine and thyroid stimulating hormone to exclude hypothyroidism. An electrocardiogram should be done in view of the high prevalence of hypertension and cardiovascular disease in obesity. Further investigations will depend on the degree of clinical suspicion of underlying (for example, Cushing's disease). The measurement of plasma leptin is not routinely indicated but may be useful in suspected cases of leptin deficiency or in severe lipodystrophy. Young patients with features of monogenic forms of obesity should be referred to a specialist center for further investigations.

## COMPLICATIONS

- *Psychosocial:* Poor self-esteem, anxiety, depression, eating disorders, social isolation.^35^
- *Endocrinal:* Insulin resistance, type 2 diabetes, early puberty.
- *Cardiovascular:* High blood pressure, high cholesterol, advanced vascular ages, early onset atherosclerosis.
- *Pulmonary:* Sleep apnea, asthma, exercise intolerance.
- *Gastrointestinal:* Fatty liver, gallstones, constipation, cirrhosis.
- *Musculoskeletal:* Blount's disease, back pain.

## MANAGEMENT

- Depends on age, severity, underlying cause
- *Infants <2 years age:* Urgent thyroid evaluation, avoid severe calorie restriction
- Children <7 years with no other health concerns, aim at weight maintenance rather than weight loss.

### Diet

Balanced diet, adequate fiber, plenty of fluids, avoid calorie dense foods.^6,22-26^

A high-fiber, low-fat diet has been shown to reduce overweight/obesity in women. High-fiber diets can reduce low-density lipoprotein cholesterol, increase postmeal satiety and decrease subsequent hunger.

The American Heart Association dietary guidelines for children recommend the inclusion of fiber-rich foods, including fruits, vegetables, whole grains and legumes. An increase in fruits and vegetables can help reduce consumption of energy-dense foods.

Encourage foods with low glycemic index (fruits, salads, whole wheat products).

Dietary factors that promote obesity include high-calorie beverages (sugared soft drinks or fruit juice), energy-dense foods (fast foods, snack foods), excess refined carbohydrates, excess dietary fat and large portion sizes. The increase in the prevalence of obesity has coincided with an increase in portion sizes of foods both inside and outside the home, which suggests that larger portions may play a role in the obesity epidemic. Nutritional factors inherent in fast food, such as low levels of dietary fiber, high palatability, high energy density, high fat content, high glycemic load and high content of sugar in liquid form, may promote excess energy intake. To outline a dietary treatment plan and to provide adequate education, counseling by a health professional with expertize in dietary management is often required. The use of a qualified and experienced health professional, preferably a registered dietitian, for dietary counseling and to implement an optimal dietary plan for achieving and maintaining a healthy body weight is recommended.

Weight loss should be slow and steady ½ kg per week/ month.

Do not allow eating in front of TV.

### Exercise

The benefits of exercise in the management of pediatric obesity are cumulative.^27-30^ Overtime, consistent exercise will result in a multitude of metabolic and physiological benefits by promoting weight loss through increased energy expenditure and possibly through inhibition of food intake. Physical activity also helps maintain a desirable weight and helps reduce risk factors for cardiovascular disease, as shown in recent studies in children. Frequent vigorous exercise periods have been shown to be associated with decreased abdominal fat in youth. Recently, strength training was shown to be an independent predictor of lower insulin resistance in children.

The compliance is better, if activity is enjoyable– swimming, dancing and sports.

Initially low impact, moderate intensity exercise should be started to avoid injuries. As fitness improves time and intensity built-up. Gyms and supervized programs useful for adolescents, stairs instead of lifts, walking for errands, etc. are helpful. Exercise for overweight children should be appropriate to their specific physiological and metabolic condition.

### Parenting Skills

Parents should be a role model.^31^ Family-based behavioral interventions are the most widely studied types of intervention, with evidence of long-term success among 8- to 12-year-old children.

Keep undesirable foods out of home, reduce frequency of eating out.

Zero calorie reward (hug, praise, sticker, fancy pen) should be given on compliance.

Limit household screen time: TV, computer games to <2 hours a day.

### New Techniques

ETIOBE: This e-health platform is an e-therapy system for the treatment of obesity, aimed at improving treatment adherence and promoting the mechanisms of self-control in patients, to obtain weight loss maintenance and to prevent relapse by establishing healthy lifestyle habits.^32^ ETIOBE is composed of three different applications, the clinician support system (CSS), the home support system (HSS) and the mobile support system (MSS). The use of new information and communication technologies (ICT) can help clinicians to improve the effectiveness of weight loss treatments, especially in the case of children and to achieve designated treatment goals.

### Pharmacological Treatment

The available pharmacological agents for weight loss include orlistat (a lipase inhibitor that prevents absorption of dietary fat from the gut) and sibutramine (an inhibitor of serotonin, norepinephrine and dopamine reuptake).^33,35-38^

While there is evidence for modest effectiveness of orlistat and sibutramine when combined with lifestyle intervention, treatment with these medications is associated with more adverse effects than lifestyle intervention alone.

Sibutramine, a serotonin nonadrenaline reuptake inhibitor enhances satiety and has been shown to be the most effective drug in treating adolescent obesity. This drug may be associated with side effects including increases in heart rate and blood pressure limiting its use in obese adolescents with higher blood pressure.

Orlistat, which is a pancreatic lipase inhibitor, acts by increasing fecal fat loss. It is associated with flatulence, diarrhea, gallbladder diseases, malabsorptive stools and requires fat-soluble vitamin supplementation and monitoring. Orlistat appears to be less effective in those who follow diets which are low in fats as is the case of many Indian diets. Metformin is a valuable adjuvant to the treatment of obese adolescents with severe insulin resistance, impaired glucose tolerance or polycystic ovarian syndrome. Pharmacotherapy should be reserved as a second line of management and should be considered only when insulin resistance, impaired glucose tolerance, hepatic steatosis, dyslipidemia or severe menstrual dysfunction persist inspite of lifestyle interventions. All patients should be given dietary, exercise and lifestyle modification instructions and counseling.

### Surgical Treatment

Limited data suggest that important comorbidities improve after bariatric surgery in adolescents, perhaps more in youth than in adults, given that most pediatric comorbidities are of shorter duration.^33-35,39^ Analysis of perioperative complications indicates that bariatric surgical procedures are generally safe, with complications that are similar to those seen in adults. Adolescent candidates for bariatric surgery should be very severely obese (defined by BMI of >40), have attained a majority of skeletal maturity (generally >13 years of age for girls and >15 years of age for boys) and have comorbidities related to obesity that might be remedied with durable weight loss. More severe elevation of BMI (>50 kg/ m^2^) may be an indication for surgical treatment in the presence of less severe comorbidities. The bariatric procedures preferred in adolescents are Roux-en-Y gastric bypass, vertical banded gastroplasty and adjustable gastric banding.

To date there are no randomized controlled trials of bariatric surgery in children.

No perioperative mortality has been detected in pediatric age groups. There are insufficient data to permit assessment of long-term risks or recidivism in young patients.

Long-term prospective studies are needed to establish the safety and efficacy of restrictive and malabsorptive procedures and to determine whether reductions in morbidity and mortality outweigh the risks of serious surgical complications and life-long nutritional deficiencies.

## CONCLUSION

Childhood obesity is a significant threat to the long-term health and well-being of Indian children. Obesity contributes to a significant burden in terms of chronic diseases, rising health care costs, and most importantly, disability and premature death. It appears that this burden will increase in the future. Thus, dentists can and should play an important role in controlling this rising epidemic.
